# The Development and Application of Vegetable Genomics Increase the Efficiency of Exploring New Gene Resources for Vegetables

**DOI:** 10.3390/ijms25136906

**Published:** 2024-06-24

**Authors:** Xi-Xiang Li, Yun-Song Lai

**Affiliations:** 1 State Key Laboratory of Vegetable Biobreeding, Institute of Vegetables and Flowers, Chinese Academy of Agricultural Sciences, Beijing 100081, China; 2 College of Horticulture, Sichuan Agricultural University, Chengdu 611100, China

Vegetables, as indispensable non-staple foods in people’s daily diet, provide a variety of essential vitamins, minerals, and other nutrients, as well as special phytochemicals, which are recognized as functional components for human nutritional balance or medicinal purposes. To effectively improve the quality and production performance of vegetables, global researchers have been working hard to understand the genetic bases and regulation mechanisms of important horticultural traits and biotic/abiotic stress tolerance.

The emergence and application of Next-Generation Sequencing (NGS) at the beginning of the 21st century, and subsequently the development of the third-generation sequencing technology represented by the platforms of Oxford nanopore sequencing (ONS) and PacBio sequencing that generate extra-long reads and therefore make genome/mRNA/ncRNA assembly much easier, greatly promote the advances in genetics and genomics of vegetable crops [1,2]. Cucumber is the first vegetable crop with a released genome whose genomic sequencing was accomplished in 2009, and henceforth, more than 30 vegetable crops have been genome-released [3]. To meet the demand of today’s bioinformatic analysis, the released genomes keep updating, e.g., hot pepper [4], tomato [5], eggplant [6,7,8], cucumber [9], water melon [10], and melon [11], by using single-molecule long reads and high-throughput chromosome conformation capture (Hi-C).

In order to follow up on the frontier research of vegetable crops, we launched this Special Issue, “Vegetable Genetics and Genomics”, at the end of 2021, which aimed to recruit both original research articles and review papers that target horticultural traits and reveal the underlying genes and genetic mechanisms by using advanced technologies of molecular genetics and multiple omics.

This Special Issue attracted researchers from 25 institutes all over the world who contributed 12 research articles and 1 review article. There are six articles about Solanaceae plants, three articles about Cruciferae plants, two articles about Cucurbitaceae plants, and one article each about garlic leaks and olives. Basically, common vegetables are of much wider research interest. The flower stalks and inflorescence of rapeseed are a very popular vegetable in East Asian countries, and processed olives are taken as a vegetable in Europe countries. Therefore, we included these two articles in this Special Issue.

Comparative transcriptome analysis is widely used to profile the expression change of gene sets and therefore identify the candidate genes controlling the target phenotypes. The wax gourd (*Benincasa hispida*) is an important Asian fruit vegetable crop. Luo et al. identified 68 small auxin-upregulated RNA (SAUR) genes in gourd and found *BhSAUR60* displayed distinct expression patterns and might play a role in fruit development [12]. Potatoes have differential performance in tuberization ability, which can be quantified by MS medium culture. Valencia-Lozano et al. found the regulated genes involved in potato microtuber development by comparing two potatoes with extreme tuberization traits; they constructed a protein–protein interaction network of related transcription factors involving light and photosynthesis, hormones, and hormone-mediated signaling pathways [13]. Olive is a fruit and vegetable crop originated in Mediterranean coast regions, and a Spanish team profiled the transcriptome change at the anthesis stage and six fruit-development stages. Combining the cytological and hormonal data, they discovered the mutual coordination of candidate transcription factors, cell cycle-regulating genes, plant hormone regulators, cell wall biosynthesis, and signaling genes in olive fruit development [14]. This article is a good example of making full use of multi-faceted supportive data.

Integrated transcriptome, metabolome, and other analytical strategies were also used to dissect certain physiological phenomena or biochemical metabolic pathways that may be involved in vegetable product quality. Radish is known for its accumulation of beneficial glucosinolates (GSLs). Li et al. profiled the differential expression of GLS pathway genes between low and high GSL content radishes and proposed the central role of MYB28 in GLS biosynthesis [15]. Zhou et al. compared five differentially colored eggplants (anthocyanin pigmented fruits, green fruits, and white fruits) and indicated the activation of early phenylpropanoid biosynthesis genes (*SmPAL*, *SmC4H*, and *Sm4CL*) was responsible for anthocyanin accumulation, while *SmF3′5′H* was the key factor for the formation of a purple color. Two transcription factors, *SmGL2* and *SmGATA26*, were also associated with anthocyanin accumulation [16]. Villa-Rivera et al. compared the expression changes of carotenoid biosynthesis genes between the fruits of 12 chili pepper accessions during the development and ripening processes. They identified the relevant transcription factor for carotenoid biosynthesis by coexpression analysis [17]. By combining comparative genomics and expression pattern analysis with VIGS, subcellular localization, yeast one-hybrid, and dual-luciferase reporter assays, Liu et al. systematically identified members of the CaMYB-related family, predicted their possible biological functions, and revealed that CaMYB37 is critical for the transcriptional regulation of capsaicin biosynthesis in peppers [18]. Coupling RNA-seq and metabolomes, Xia et al. identified garlic odor compounds, free amino acids, and sugars from wild Chinese chives and compared them with cultivated Chinese chives. They cloned the structural genes of garlic odor compounds in wild Chinese chives and found that a deletion in the FMO gene resulted in an impaired protein sequence and the subsequently changed garlic odor compound [19].

Resistance to biotic and abiotic stresses is a major vegetable breeding target trait. Xiao et al. constructed a ChIP-Seq library to profile the transcriptionally targeted genes of Teosinte branched 1/cycloidea/proliferating cell factor (TCP) during the infection of bacterial wilt in eggplant and showed that SmTCP7a positively regulated bacterial wilt that was caused by *Ralstonia solanacearum* [20]. Yan et al. identified 65 glyoxalase (GLY) genes in the rapeseed genome and clarified its possible regulation role in freezing tolerance in Arabidopsis [21]. Compared with molecular markers used in conventional gene/QTL mapping, genomic sequencing-based genotyping is a more convenient and effective alternative. Liu et al. conducted Genome-Wide Association Studies (GWASs) by re-sequencing 220 cucumber core accessions and found that a total of seven loci were associated with salt tolerance in cucumber seedlings and finally predicted five candidate genes controlling salt tolerance [22].

Cytoplasmic male sterility (CMS) is widely used to produce hybrid seeds in vegetable crops, and great efforts have been made to identify male sterility and restorer genes. Ogura is a well-known CMS source found in Japanese radishes, but now it has been transferred into many other Cruciferae crops. Ren et al. well summarized the explosion and utilization of Ogura CMS sources in Cruciferae crops [23]. This is the only review article included in this Special Issue. One research article identified a new candidate gene for restorer-of-fertility (Rf) in capsicum pepper. The single dominant gene *CaRf_HZ_* was mapped in a QTL of 533 kb through bulked segregant analysis (BSA)-sequencing [24]. Male sterility is widely used to produce hybrid F1 seeds, and these studies contribute to exploring and developing new male sterility resources based on conventional ones.

To sum up, the articles compiled in this Special Issue provide valuable and novel knowledge of the genetic and molecular mechanisms behind certain important traits of vegetable crops. Nevertheless, further laboratory experiments should be carried out in the future, which can provide more biological evidence to confirm the proposed gene function. In addition, there are hundreds of vegetables on the market, and vegetable science has an interesting scope of thousands of plant species, which is much more than other agricultural research fields like crop science, pomology, ornamental plants, and so on. A lot of vegetable crops have a lot of interesting agronomic traits that need to be clarified. It is worthwhile to expect that the application of advanced genetic/genomic strategies and technology can accelerate the process. We have the support of the editorial office to launch a new Special Issue focusing on the same research theme, entitled “Vegetable Genetics and Genomics 2.0”. We will make a continuous effort on this topic and sincerely invite researchers from all over the world to contribute their latest research results.
